# Forgotten joint score associated with prosthesis weight in cementless total hip arthroplasty: a prospective clinical study

**DOI:** 10.3389/fsurg.2024.1210668

**Published:** 2024-07-30

**Authors:** Huiliang Zeng, Ping Li, Wenjun Feng, Ke Jie, Jinlun Chen, Jianchun Zeng, Xicong Chen, Guanming Zhou, Haitao Zhang, Yirong Zeng

**Affiliations:** ^1^ The First Clinical College, Guangzhou University of Chinese Medicine, Guangzhou, Guangdong, China; ^2^ Department of Joint Surgery, Foshan Hospital of Traditional Chinese Medicine, Foshan, Guangdong, China; ^3^ Department of Anorectal, Guangdong Provincial Hospital of Integrated Traditional Chinese and Western Medicine, Foshan, Guangdong, China; ^4^ Department of Joint Surgery, The First Affiliated Hospital of Guangzhou University of Chinese Medicine, Guangzhou, Guangdong, China; ^5^ Longhua Hospital, Shanghai University of Traditional Chinese Medicine, Shanghai, China

**Keywords:** total hip arthroplasty, prosthesis weight, removed bone weight, forgotten joint score, clinical study

## Abstract

**Background:**

This prospective study aimed to investigate the influence of weight difference between implanted prosthesis and removed bone in cementless total hip arthroplasty (THA) on hip awareness and patient-reported outcomes.

**Methods:**

A total of 48 patients (56 hips) who underwent primary THA were prospectively enrolled. Implanted prosthesis and removed bone were weighed intraoperatively. Forgotten Joint Score (FJS) and Western Ontario and McMaster Universities (WOMAC) scores were obtained before and at 1 and 3 months after surgery. Patients were divided into groups A, B, and C according to the percentile of the weight difference.

**Results:**

The mean weight difference of the implanted prosthesis and removed bone was 117.97 ± 47.35 g. A negative correlation was found among the weight differences of the three groups and 1- and 3-month postoperative FJS (correlation coefficients, −0.331 and −0.734, respectively). A positive correlation was found among the weight difference of the three groups and 3-month postoperative WOMAC (correlation coefficient, 0.403). A significant difference in 3-month postoperative FJS and WOMAC scores was found among the three groups. The mean 3-month postoperative FJS (79.00) of group C was significantly lower than that of group A (93.32) (*P* < 0.05). The mean WOMAC score (15.83) of group A was significantly lower than that of group C (23.67) (*P* < 0.05).

**Conclusion:**

The implanted prosthesis is larger than the removed bone in cementless THA. The weight difference is negatively correlated with hip function. The weight difference should be minimized to achieve optimal hip joint awareness.

## Introduction

Total hip arthroplasty (THA) has been accepted as an effective procedure in the treatment of advanced hip arthrosis (such as osteonecrosis of the femoral head (ONFH) and hip osteoarthritis (HOA) with a nearly 95% and 80% (1) survivorship at 10 years and 25 years, respectively. As THA is frequently performed worldwide, the demand for THA is increasing due to the aging population. The estimated annual number of primary THAs in the United States by 2030 is 572,000 (2). Moreover, various studies confirmed that performing THA with appropriate surgical techniques and excellent prosthesis survivorship could relieve pain, improve function, and achieve high patient satisfaction (3–5).

Although most THA patients were satisfied with the procedure, data showed that 3%–16% of patients (6, 7) were unhappy because of persistent postoperative pain and impaired function (8). However, in our outpatient follow-up, we observed that some patients with excellent hip function complained about the “heavy” feeling and could not “forget” the artificial hip in the early stage after surgery. When reviewing relevant literature, we found that “joint awareness” is getting more and more attention (9–17).

Joint awareness refers to how the patient felt about the artificial joint in daily life, which represents the extent of forgetting the arthroplasty and is evaluated with the forgotten joint score (FJS) (18).

Thus, the ultimate goal is to ensure utmost patient satisfaction in total joint arthroplasty (TJA). Furthermore, sex (18), location (knee vs. hip) (18), prosthesis types [posterior stabilized total knee arthroplasty (TKA) vs. patellofemoral resurfacing arthroplasty] (10), pain, and quadriceps strength (19) have been proven to be influencing factors of joint awareness. Gibon et al. (20) suggested that the total weight of implants and cement is heavier than that of the removed bone and soft tissues in TKA, despite the different brands of the prosthesis. Thus, they speculated that prosthesis weight may play an important role in postoperative joint awareness following TJA.

Given our observation and the above findings and speculation, we contemplate whether the weight of implants affects patients’ hip awareness in THA. To our knowledge, no study has explored the prosthesis weight and its association with patient-reported outcomes (PROs). Therefore, in this study, we aimed to obtain the weight difference between the implanted implants and the removed bone tissues to determine whether the weight difference affects postoperative joint awareness in THA.

## Methods

### Study design, inclusion and exclusion criteria

This prospective study included patients who underwent unilateral or bilateral THAs in the 1st Affiliated Hospital of Guangzhou University of Chinese Medicine from April 2018 to October 2018. The study was approved by the Ethics Committee of the hospital (NO. ZYYECK [2018]012) and then registered at a clinical trial registry (http://www.chictr.org.cn) with the registry number ChiCTR1800015462. All patients were informed about the details of the study, and written consent was obtained. The research data and outcomes were blinded to all patients.

Patients who underwent THA for advanced ONFH or HOA were included. Patients who had undergone surgery or had a traumatic history of surgical hips failed to complete or refused our follow-up plan, and had incomplete data were excluded. Unclear tears or unqualified x-rays have been excluded. The demographics (name, age, sex), body mass index (BMI), medical history of the affected hip, education level, and preoperative and postoperative anteroposterior pelvic x-rays were recorded. Patients whose postoperative leg-length discrepancy (LLD) was more than 10 mm and/or patients whose postoperative absolute femoral offset (FO) difference of the bilateral hip was more than 4 mm were removed for further analysis.

### Surgical technique

All surgeries were performed by the same surgeon (YRZ) with a standard posterolateral approach, and the capsule was spared. The same surgical technique and prosthesis brand were used (LINK Orthopaedics, Germany). Spinal analgesia was routinely used. The implants used were LINK cementless prosthesis (Waldemar Link, Hamburg, Germany). The hip system consisted of an LCU femoral stem design and three types of liner, namely, ceramic on ceramic, polyethylene on ceramic, and metal on polyethtlene. All operations are performed by the same surgeon with mature surgical skills.

### Main observations

An experimental electronic scale with an accuracy setting of 0.01 g was used to measure the weight of the resected bone tissues and implanted prosthesis.

**Figure 1:**
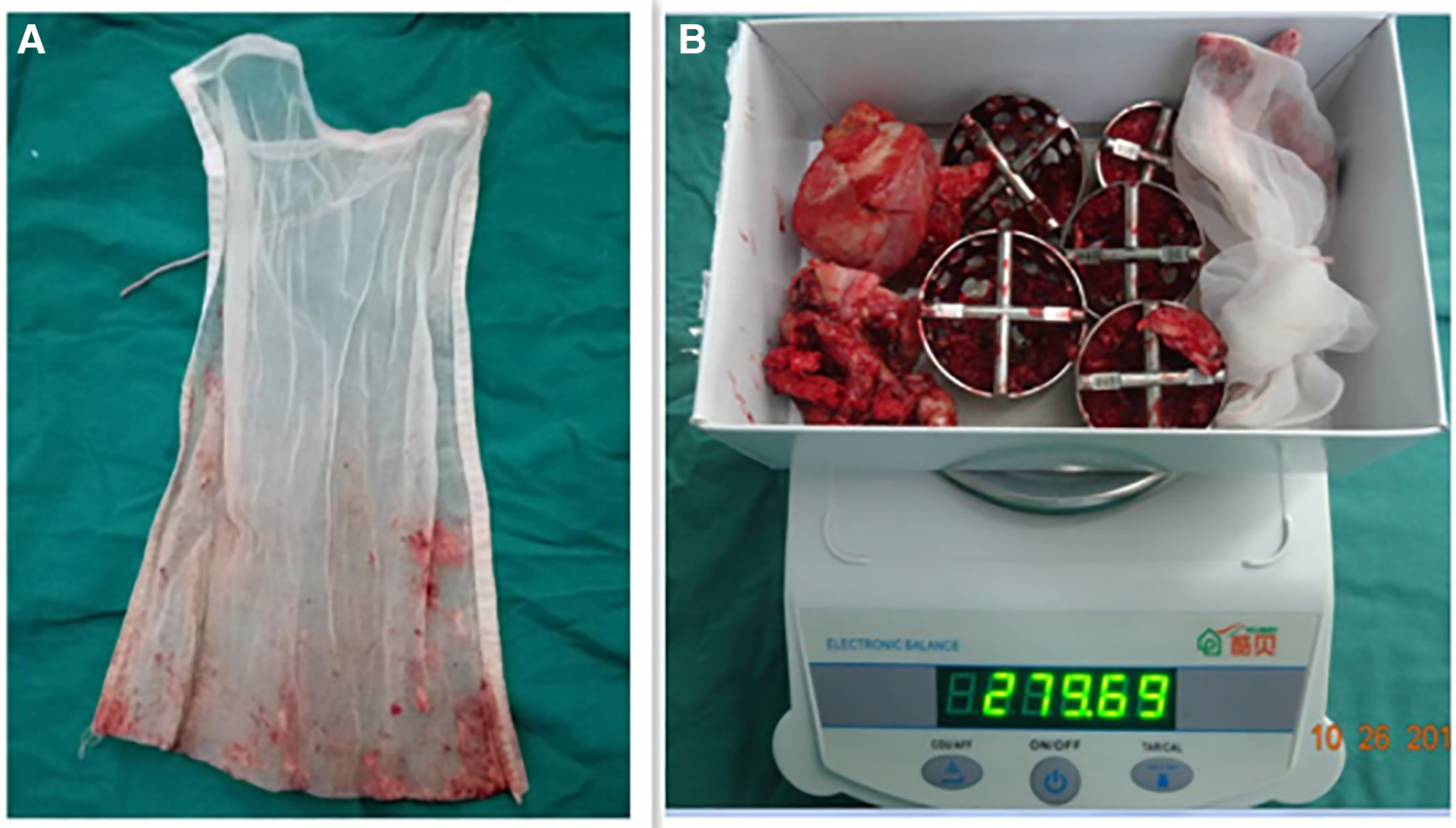
Medullary bone collected by a 22 × 30-cm 100-mesh filter bag. (**A**) Weighing the removed bone in the package, acetabular honing tool, and filter bag (**B**).

**Figure 2:**
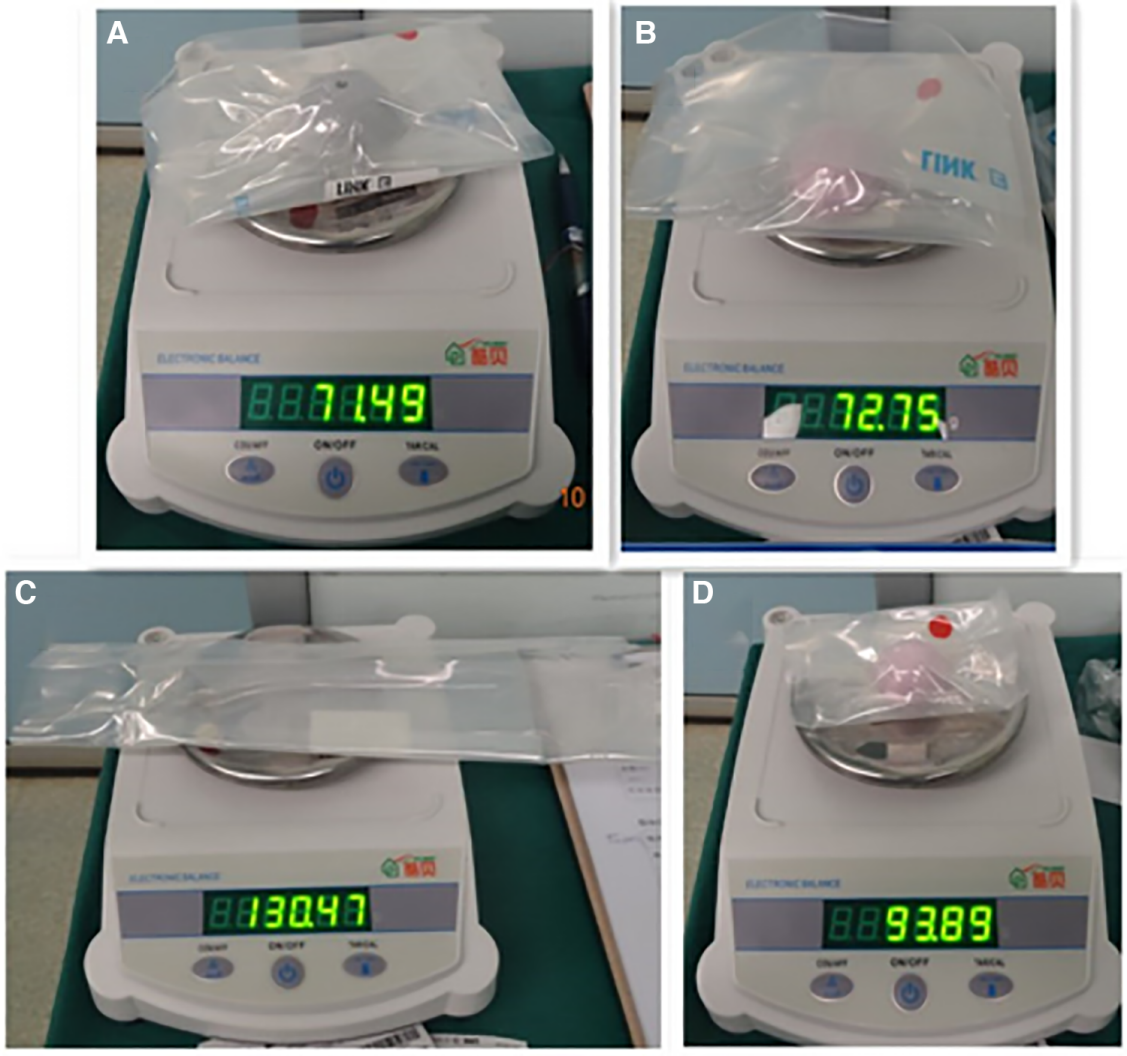
Weighing the THA prosthesis with its inner bag: cup (**A**), liner (**B**), femoral stem (**C**), and femoral head (**D**) components. THA, total hip arthroplasty.

(1) Bone tissue weight: The removed bone tissues include the femoral head, femoral neck, bone from the acetabular and femur medullary cavities, and osteophytes around the hip. A prosthetic package and a 30 cm (length) × 22 cm (width) 100-mesh filter bag were prepared and then weighed. The femoral head, neck, and osteophytes were placed in the package. All surgeries used the same acetabular reamer and were weighed before surgery. Acetabular bone tissues were gathered using the reamer. Subsequently, 4 ml of heparin solution and 50 ml of 0.9% sodium chloride solution were added to the suction bag before surgery and shaken evenly to keep the blood drawn from the surgical site from condensing. The mesh bag contained the bone from the femur medullary cavity. Bone weight was measured by weighing the above collections, and subtracting the weight of the package, acetabular honing tool, and filter bag (Figure 1).
(2) Prosthesis weight: the implanted components include the acetabular cup, liner, femoral head, and stem. The inner package was weighed together with the prosthesis before unpacking (Figure 2). Then, only the packages were weighed after the prostheses were implanted. The prosthesis weight was the weight of the bag subtracted from the weight before unpacking.

### Secondary observations

**Figure 3:**
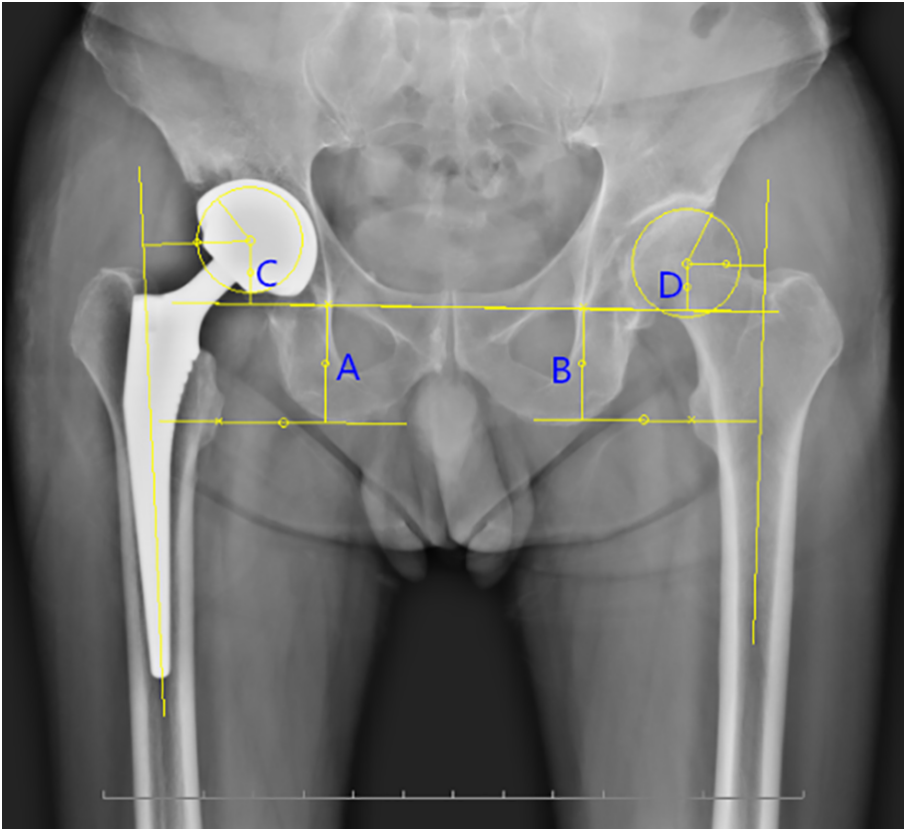
Measurement of the LLD and FO in the anteroposterior radiograph. LLD is defined as the absolute value of the sum of (A,C) minus the sum of (B,D). FO is the vertical distance from the rotation center of the femoral head to the long axis of the femur. FO, femoral offset; LLD, limb-length discrepancy.

The LLD and FO were measured using Digimizer Version 4.3.4 (MedCalc Software bv, Osbend, Belgium). (1) LLD: the measurement (21) was performed in the anteroposterior radiograph (Figure 3). First, a line was drawn through the midpoint of the bilateral small rotor horizontal to the line connected to the distal point of the bilateral teardrop; by idealizing the femoral head into a circle to determine its rotation center, another line was drawn through the femoral head rotation center and parallel to the line of the bilateral teardrop. Finally, four vertical lines were drawn: Lines A and B indicate the distance from the distal point of the teardrop to a small rotor on the surgical side and the healthy side, respectively. Lines C and D indicate the distance from the teardrop to the femoral head rotation center on the surgical side and healthy side, successively. LLD is defined as the Line A minus B (Figure 3). (2) Bilateral FO difference: FO is the vertical distance from the rotation center of the femoral head to the long axis of the femur. According to the Nunn standard, FO is measured as the vertical distance from the bilateral femoral head rotation center to the ipsilateral femoral long axis. The difference between the bilateral vertical distances is the FO difference.

### Grouping method

Weight difference was calculated as the difference between the weight of the implanted prosthesis and the weight of the removed bone. It is divided into minor (group A), medium (group B), and major (group C) by 33% and 67% of the weight difference percentile.

### Follow-up

All patients filled the FJS and WOMAC forms for every hip preoperatively and at 1 month and 3 months postoperatively.

### Statistical analysis

SPSS 24.0 (IBM Corp., Armonk, NY, USA) was used for data analysis. The chi-square test and Fisher's exact test were used to analyze sex, education level, diagnosis, and side among the three groups. One-way analysis of variance (ANOVA) was used to analyze age, BMI, length of hospital stay, LLD, FO difference, preoperative and postoperative FJS, and WOMAC score, and the least significant difference (LSD) method was used for comparison. Spearman correlation analysis was used to determine the relevance between the weight difference of the three groups and PRO. *P* < 0.05 was considered statistically significant.

## Results

Fifty patients (56 hips) were enrolled in the study, and 2 patients (2 hips) were lost to follow-up at 3 months postoperatively. A total of 48 patients (56 hips), with an average age of 54.71 ± 12.48 (range, 27–85) years were included in the study. The average weight of the removed bone was 142.87 ± 34.07 g (range, 86.05–231.33 g), the average weight of the implanted hip prosthesis was 260.78 ± 53.83 g (range, 155.23–361.69 g), and the average weight difference was 117.97 ± 47.35 g (Tables 1, 2).

**Table 1:** Descriptive statistics of research data.

	*N*	MV	SD	Minimum	Maximum
Age (yr)	56	54.71	12.48	27	85
BMI (kg/m^2^)	56	23.51	3.15	17.92	31.93
Hospital time (day)	56	9.27	2.28	4	14
Postoperative	56	0.15	0.05	0.02	0.27
FO difference (cm)					
Postoperative	56	0.06	0.09	0	0.3
LLD (cm)					
Bone weight (g)	56	142.87	34.07	86.05	231.33
Prosthesis	56	260.78	53.83	155.23	361.69
Weight (g)					
Weight difference (g)	56	117.91	47.35	18.46	236.81

*N, number; MV, mean value; SD, standard deviation.

**Table 2:** Descriptive statistics of research data.

		Frequency	Percentage
Sex	Male	23	41.1
	Female	33	58.9
Education	Illiterate	4	7.1
	Primary	12	21.4
	Junior	30	53.6
	High	7	12.5
	College	2	3.6
	Bachelor	1	1.8
Diagnosis	OA	36	64.3
	ONFH	20	35.7
Side	Left	30	53.6
	Right	26	46.4

OA, osteoarthritis; ONFH, osteonecrosis of the femoral head.

According to the percentile of the weight difference, the median weight was 121.52 g, the weight difference at the 33 percentile (P33) was 94.68 g, and the weight difference at the 67 percentile (P67) was 137.84 g. The patients were divided into three groups according to the P33 and P67 method: group A, less than 94.68 g (*n* = 18, 32.14%); group B, between 94.68 and 137.84 g (*n* = 20, 35.72%); and group C, more than 137.84 g (*n* = 18, 32.14%) (Table 3).

**Table 3:** Percentile statistics of weight difference.

Percentile	25	33	50	67	75
WD (g)	86.09	94.68	121.52	137.84	148.63

WD, weight difference.

The results of the chi-square test and Fisher's exact test analysis (Table 4) showed no significant difference in education level, diagnosis, and operation side among the three groups (*P* > 0.05). However, significant differences in sex were noted among the groups (*P* < 0.05). The pairwise comparison revealed that the proportion of male patients in group C at 72.2% was significantly higher than that in group A at 22.2% and in group B at 30% (*P* < 0.05), which indicated that male THA patients have a relatively larger weight difference.

**Table 4:** Statistical analysis of qualitative demographics.

	Group A (*n* = 18)	Group B (*n* = 20)	Group C (*n* = 18)	X^2^	*P*
Sex					
Male	4 (22.2%)	6 (30.0%)	13 (72.2%)*^,^^#^	10.872	0.004
Female	14 (77.8%)	14 (70.0%)	5 (27.8%)		
Education					
Illiterate	3 (16.7%)	1 (5.05)	0	–	0.343
Primary	5 (27.8%)	3 (15.0%)	4 (22.2%)		
Junior	9 (50.0%)	10 (50.0%)	11 (61.1%)		
High	1 (5.6%)	5 (25.0%)	1 (5.6%)		
College	0	1 (5.0%)	1 (5.6%)		
Bachelor	0	0	1 (5.6%)		
Diagnosis					
OA	13 (72.2%)	13 (65.0%)	10 (55.6%)	1.096	0.578
ONFH	5 (27.8%)	7 (35.0%)	8 (44.4%)		
Side					
Left	10 (55.6%)	11 (55.0%)	9 (50.0%)	0.137	0.934
Right	8 (44.4%)	9 (45.0%)	9 (50.0%)		

OA, osteoarthritis; ONFH, osteonecrosis of the femoral head.

* Indicates that other groups are compared with group A, *P* < 0.05.

^#^ Indicates that other groups are compared with group B, *P* < 0.05.

The results of the one-way ANOVA and LSD method comparison showed (Table 5) no significant difference in BMI, hospital stay, LLD, and FO difference among the three groups (*P* > 0.05), but significant differences in age were observed (*P* < 0.05). After a pairwise comparison, the mean age of group C (50.33 years) was significantly lower than that of group A (60.67 years) (*P* < 0.05). This indicated that younger THA patients have larger weight differences.

**Table 5:** Statistical analysis of quantitative demographics.

	N	Age (yr)	BMI (kg/m^2^)	Hospital time (day)	FO difference (cm)	LLD (cm)
Group A	18	60.67 ± 13.81	24.08 ± 2.42	9.28 ± 2.22	0.15 ± 0.04	0.05 ± 0.10
Group B	20	53.30 ± 11.35	24.08 ± 3.24	9.65 ± 2.58	0.15 ± 0.07	0.07 ± 0.09
Group C	18	50.33 ± 10.47*	22.31 ± 3.52	8.83 ± 2.04	0.16 ± 0.05	0.08 ± 0.09
F		3.592	1.999	0.597	0.282	0.383
*P*		0.034	0.145	0.554	0.755	0.684

* Indicates that other groups are compared with group A, *P* < 0.05 ($\bar{x}$ ± S). BMI, body mass index; FO, femoral offset; LLD, limb-length discrepancy.

On the Spearman correlation analysis (Table 6), the weight difference has a significant correlation with the 1-month and 3-month postoperative FJS (*P* < 0.05 or 0.01; correlation coefficients, −0.331 and −0.734, respectively), which means that weight difference was negatively correlated with the 1-month and 3-month postoperative FJS. This indicates that the greater the weight difference, the lower the FJS at 1 month and 3 months after surgery, the higher the degree of hip discomfort on the operation side, and the worse the hip awareness, the harder it is to attain “forgotten hip.” There was a significant positive correlation between the weight difference and the 3-month postoperative WOMAC score (*P* < 0.05 or 0.01; correlation coefficient, 0.403), which indicates that the greater the weight difference, the worse the postoperative patients’ function.

**Table 6:** Correlation analysis between weight difference and patient-reported outcomes between the three groups.

		Group	1-month post-op FJS	3-month post-op FJS	1-month post-op WOMAC score	3-month post-op WOMAC score
Group	Correlation coefficient significance (two-tailed)	1.000	NA	NA	NA	NA
		NA	NA	NA	NA	NA
1-month post-op FJS	Correlation coefficient significance	−.331^b^	1.000	NA	NA	NA
	(Two-tailed)	0.013	NA	NA	NA	NA
3-month post-op FJS	Correlation coefficient significance	−0.734^a^	0.539^a^	1.000	NA	NA
	(Two-tailed)	0.000	0.000	NA	NA	NA
1-month post-op WOMAC	Correlation coefficient significance	−0.011	−0.344^a^	−0.255	1.000	NA
	(Two-tailed)	0.936	0.009	0.058	NA	NA
3-mont post-op WOMAC	Correlation coefficient significance	0.403^a^	−0.370^a^	−0.655^a^	0.500^a^	1.000
	(Two-tailed)	0.002	0.005	.000	0.000	NA

FJS, forgotten joint score; post-op, postoperative; WOMAC, Western Ontario and McMaster Universities Osteoarthritis Index score.

^a^ At the 0.01 level (two-tailed), the correlation is significant.

^b^ At the 0.05 level (two-tailed), the correlation is significant.

Comparing the preoperative and postoperative PRO of the three groups using one-way ANOVA and LSD method (Table 7), no significant difference in 1-month postoperative FJS was found among the three groups (*P* > 0.05), but there was a significant difference in 3-month postoperative FJS and WOMAC scores (*P* < 0.05). On the pairwise comparison, the mean 3-month postoperative FJS (79.00) in group C was significantly lower than that in group A (93.32) (*P* < 0.05), which means that the hip awareness of patients with a weight difference of equal to or less than 94.68 g was better than that of patients with larger weight difference. The mean 3-month postoperative WOMAC score (15.83) in group A was significantly lower than that in group C (23.67) (*P *< 0.05), indicating better function in group A than in group C.

**Table 7:** Statistical analysis of postoperative patient-reported outcomes.

	*N*	1-month post-op FJS	3-month post-op FJS	1-month post-op WOMAC score	3-month post-op WOMAC score
Group A	18	70.70 ± 14.05	93.32 ± 2.27	52.39 ± 21.29	15.83 ± 6.65
Group B	20	65.50 ± 18.55	87.59 ± 6.71	52.50 ± 19.40	20.35 ± 9.89
Group C	18	57.24 ± 19.21	79.00 ± 10.91*	52.56 ± 14.47	23.67 ± 10.20*
F		2.723	16.722	0.000	3.370
*P*		0.075	0.000	1.000	0.042

FJS, forgotten joint score; post-op, postoperative; WOMAC, Western Ontario and McMaster Universities Osteoarthritis Index score.

* Indicates that other groups are compared with group A, *P* < 0.05.

## Discussion

Many published studies have detected the factors affecting PROs after THA, such as preoperative gait (22), expectation (23), surgical technique (24) [including approach (25–28), surgeon experience (29), LLD (30, 31), FO (32)], postoperative rehabilitation (33), metal allergy (34), and alkaptonuria (35), etc. Nevertheless, few studies have focused on joint awareness of THA and its influencing factors.

To our knowledge, this is the first study obtaining data about weight and weight difference between implanted prosthesis and removed bones in cementless THA. The mean weight of the prosthesis is about twice that of the removed bone (142.87 g). Most of the THA cases with larger weight differences were found in younger and male patients. We also found that weight difference was negatively correlated with postoperative joint awareness, which means that the greater the difference, the worse was the hip awareness. Patients with a cutoff difference value equal to or less than 94.68 g showed better hip awareness and function than other patients.

To minimize the bias of other possible factors, the cases analyzed were screened, an experienced surgeon accomplished all THAs using the same technique, and patients were rehabilitated under a uniform plan. Patients with postoperative LLD more than 10 mm and/or absolute value of bilateral FO difference less than 4 mm were excluded from the study because previous studies have confirmed that when LLD is equal to or less than 10 mm and/or bilateral FO difference is equal to or less than 4 mm, the effect on clinical outcomes is minimal (36).

Currently, no study has reported factors that influence the weight difference between implanted prosthesis and removed bone. We attributed this to the following aspects. (1) Prosthesis-related factor: prosthesis weight may differ in size, interface, and brands, and whether cement and cementless implants were used. Large components are heavier than medium or small ones with the same brand, which might increase the weight difference. (2) Diagnosis and stage of disease factors: Osteophyte formation is larger in HOA patients than in ONFH patients, and Kellgren-Lawrence IV osteoarthritis (OA) is more severe than that of grade III in HOA. Thus, the amount of bone removed intraoperatively increased, and the weight difference decreased. (3) Surgeon experience: when preparing the acetabular side in THA, some surgeons prefer to completely remove osteophytes surrounding the hip, while other surgeons do not, which may lead to weight difference. In this study, the surgeon thoroughly removed the osteophytes around the acetabular rim of all patients. Osteophytes around the acetabulum may have some impact on the later hip function (37, 38). (4) Bone condition: patients with osteoporosis caused by advanced age, post menstruation, corticosteroid use, and autoimmune system diseases have lower weight of removed bone than those with good bone quality, which may increase the weight difference. For other aspects, such as bone collection, some tiny bone debris that could not be collected may affect the accuracy of the removed bone weight. Thus, in this study, one researcher (HL Zeng) was specifically responsible for intraoperative bone collection.

Interestingly, our results showed that younger and male patients predominated in the larger weight difference group. Studies about Chinese proximal femoral morphology confirmed that the medullary cavity of men is significantly larger than that of women, and the position of the isthmus is lower in men. As a result, male THA patients need larger femoral stem components, causing larger weight differences. Yigang et al. also found a widening of the proximal femoral medullary cavity as people age, especially in women. However, Qin et al. (39) thought that age has little effect on the width of the cavity. We speculated that there was less osteoproliferation around the hip in young patients, especially in patients with HOA, which decreased the amount of the removed bone, causing a larger weight difference.

In this study, we chose the FJS and WOMAC scores to evaluate hip awareness and function outcomes, respectively. A previous study validated that FJS has no “ceiling effect,” which is commonly found in many PRO tools (40), and has a great discriminatory ability to find patients’ subtle discomfort from most of the satisfactory results (41). High scores mean a high degree of forgetting the artificial joint, which also means a low degree of awareness. In our study, we found a negative correlation between the weight difference and 1- and 3-month postoperative FJS, with correlation coefficients of −0.331 and −0.734, respectively. This indicates that the greater the weight difference, the lower the 1-month and 3-month postoperative FJS, the higher the hip awareness, and the harder it is to realize a “forgotten hip.” The WOMAC score is the most commonly used tool to measure PRO. Higher WOMAC scores mean worse function outcomes. Our results showed that the weight difference was positively correlated with the 3-month postoperative WOMAC score (correlation coefficient 0.403), which means that greater weight difference was related to worse functional outcomes. Matsumoto et al. found that FJS was correlated with the WOMAC score (42), which is consistent with our findings.

This study has the following limitations. First, the study had a small sample size; thus, the weight difference value is mostly distributed in the range of 100–200 g, which may cause bias in the grouping method. Second, the follow-up period was short; all cases in this study were only followed for 3 months after surgery. Achieving “forgotten hip” may take longer. However, based on the advanced technique and the application of fast-track surgery, most THA patients are expected to reach good function in the early stages following surgery (43). Third, there is still an error in the bone collection method. Although one researcher collected the removed bones of all patients, the bone debris from the saw when sawing the femoral head, bone debris was stuck on the drape, and bone debris during the operation was difficult to collect by hand. Although small, there may be bias in our research results; thus, further studies should improve bone collection methods. Finally, we did not validate a rational range of weight difference, optimum joint awareness, and function, as the weight difference in this study was equal to or less than 94.68 g, which needs further verification.

As most THAs could realize good to excellent outcomes, joint awareness should be an important part of patient satisfaction and should receive more attention. Based on our results, weight difference is a key factor of joint awareness in THA. Surgeons and implant manufacturers should focus on prosthesis weight and endeavor to minimize or even eliminate the weight difference between the prosthesis and the bone, making the “equal weight replacement” possible.

## Conclusion

In this study, the prosthesis weight is greater than the removed bone weight in cementless THA, and this weight difference is negatively correlated with hip awareness. Therefore, prosthesis weight is an important factor in postoperative hip awareness and function outcome in cementless THA. The difference between the weight of the implanted hip prosthesis and the removed bone should be minimized to achieve optimal hip joint awareness.

## Data Availability

The original contributions presented in the study are included in the article/Supplementary Material. Further inquiries can be directed to the corresponding author.
